# A Small Cellulose-Binding-Domain Protein (CBD1) in *Phytophthora* is Highly Variable in the Non-binding Amino Terminus

**DOI:** 10.1007/s00284-017-1315-x

**Published:** 2017-07-26

**Authors:** Richard W. Jones, Frances G. Perez

**Affiliations:** Genetic Improvement of Fruits and Vegetables Laboratory, USDA-ARS, 10300 Baltimore Avenue, Beltsville, MD 20705 USA

## Abstract

The small cellulose-binding-domain protein CBD1 is tightly bound to the cellulosic cell wall of the plant pathogenic stramenopile *Phytophthora infestans*. Transgene expression of the protein in potato plants also demonstrated binding to plant cell walls. A study was undertaken using 47 isolates of *P*. *infestans* from a worldwide collection, along with 17 other *Phytophthora* species and a related pathogen *Plasmopara halstedii*, to determine if the critical cell wall protein is subject to amino acid variability. Within the amino acid sequence of the secreted portion of CBD 1, encoded by the *P*. *infestans* isolates, 30 were identical with each other, and with *P*. *mirabilis*. Four isolates had one amino acid difference, each in a different location, while one isolate had two amino acid substitutions. The remaining 13 isolates had five amino acid changes that were each in identical locations (D17/G, D31/G, I32/S, T43/A, and G50/A), suggesting a single origin. Comparison of *P*. *infestans* CBD1 with other *Phytophthora* species identified extensive amino acid variation among the 60 amino acids at the amino terminus of the protein, and a high level of conservation from G61, where the critical cellulose-binding domain sequences begin, to the end of the protein (L110). While the region needed to bind to cellulose is conserved, the region that is available to interact with other cell wall components is subject to considerable variation, a feature that is evident even in the related genus *Plasmopara*. Specific changes can be used in determining intra- and inter-species relatedness. Application of this information allowed for the design of species-specific primers for PCR detection of *P*. *infestans* and *P*. *sojae*, by combining primers from the highly conserved and variable regions of the CBD1 gene.

**Electronic supplementary material:**

The online version of this article (doi:10.1007/s00284-017-1315-x) contains supplementary material, which is available to authorized users.

## Introduction

Cellulose-binding domains (CBD), found within the larger class of protein structural patterns known as carbohydrate binding modules (CBM), facilitate binding to target substrates. Fungal saprophyte-encoded CBDs are commonly found attached, by a serine, threonine-rich linker, to the amino or carboxy-terminus of extracellular, cellulolytic, and xylanolytic enzymes [3, 22, 24, 26]. Pathogenic fungi generally do not have CBDs associated with their cellulolytic enzymes unless the enzymes are deployed during a necrotrophic phase [40–42]. In addition to aiding the binding of enzymes, the CBDs can have the ability to dissociate cellulose microfibrils independently, as shown with CBDs produced in protein expression systems [9, 15, 34].

*Phytophthora* species are hemibiotrophic plant pathogens that also generally lack a CBD on their cellulolytic enzymes [6, 7, 32]; however, they are one of the few organisms that produce CBDs as discrete proteins [12, 18, 28]. The *Phytophthora* CBD-containing proteins generally harbor the binding domains at the carboxy terminal region of the protein, while the function of the amino half of the proteins remains undefined. Most likely, these proteins are involved in the development of the cellulose-containing cell walls of *Phytophthora*, and the undefined regions may interact with other carbohydrates or with other proteins in the cell wall. There is currently evidence for expression of three types of proteins, harboring a single CBD (CBD1), two CBDs (CBD4), and two CBDs plus a lectin-binding domain (CBD5 or CBEL) while other genomic sequences harboring CBD motifs have not yet been shown to be expressed [18]. The CBD1 protein from *P*. *infestans* has been shown to be cell-wall-bound, and it has been shown to bind to plant cell walls when expressed in transgenic potato plants [19]. In transgenic potatoes, the binding alters the expansion of ovary cell walls, allowing for fertility restoration. The degree of divergence in CBD sequence that would still allow or further enhance this response remains to be studied.

While many extracellular proteins from *Phytophthora* are extensively duplicated [6, 7, 32], the CBD-encoding genes are single copy. The single copy feature, along with providing a critical function in cell walls, suggests limited variation; however, there have not been any studies addressing the degree of conservation in amino acid sequence of CBDs within and between species of *Phytophthora*. We have focused on CBD1 and compared conservation of sequence in a worldwide collection of *P. infestans* (USDA-ARS, Beltsville, MD). Conservation of sequence was also assessed for a divergent set of *Phytophthora* species, and the related biotrophic downy mildew *Plasmopara halstedii*. Application of this information was made in development of CBD1-specific primers for *Phytophthora* detection.

## Materials and Methods

### Isolation and Identification of Sequences Encoding CBD 1

Total genomic DNA was isolated from liquid grown cultures of *Phytophthora infestans* and *P. mirabilis* using a Qiagen Plant DNeasy kit. Preliminary screening indicated that the CBD1-encoding gene (EU179903) could be amplified from different isolates of *P. infestans* using the primer pair CBD1f (ATGACCTCGTTGCGACTCCTGG) and CBD1r (CTAGAGCTCCAGTCGAATGAC), amplifying the full coding region. The PCR reaction mix consisted of 20 pmol primer, 50 ng DNA, and GoTaq polymerase (Promega) in a total volume of 40 microliters. The PCR amplification consisted of one cycle of 96 C for 2 min, followed by 30 cycles of 52 C for 15 s and 72 C for 45 s, then a final cycle of 72 C for 5 min.

The amplified products were cloned using a TOPO TA cloning kit (Invitrogen). Sequencing was performed commercially (Macrogen USA) using M13 forward and reverse primers. Overall, single colonies were used for sequencing since the purpose of the study was to show general variations in CBD 1, using a large number of individual isolates (Online Resource 1). Allelic variation was not determined for each isolate where CBD1 was cloned from, and allelic variation could not be determined when using GenBank sequences.

For in silico identification of CBD1 proteins from other *Phytophthora* species and *Plasmopara,* we conducted tblastn searches of NCBI databases using the specific CBD1 *Phytophthora* cellulose-binding domain sequence (GVRAWAQCGGLYYLGKTKCQQHTFCKQLSEFISVC).

### Alignments and Phylogeny

Nucleotide sequences were translated using WebMap (pga.mgh.harvard.edu) and signal peptides were identified using SignalP 4.1 (cbs.dtu.dk/services) [33]. Nucleotide and amino acid sequences were compared after removal of the signal peptide sequence. Alignment was performed using Clustal Omega 1.2.2 (ebi.ac.uk/Tools/msa/clustalo) [37] and amino acid-based phylogeny was assessed using Geneious 8.1.7 (geneious.com) [20]. Glycosylation patterns were assessed using NetNGlyc 1.0 and DictyOGlyc 1.1 (cbs.dtu.dk/services) [13]. Amino acid-based phylogeny was generated using MEGA6.0 [10, 35, 38].

### Application of CBD 1 Primers for Species Detection and Identification

For PCR detection and species discrimination, primers used included *P. infestans* forward (PiF TCTAACCTCCGGAACGGCGAC), *P. infestans* reverse (PiR TAGAGCTCCAGTCGAATGACT), *P. sojae* forward (PsF TCCAACCTCCGAAACTCGATCCATC), *P. sojae* reverse (PsR TACAGCTCGAGCCGGATGACC), and CBD reverse-common (CBD-Rc GTGTGCTGCTGGCACTTGGTCT). Primer specificity was tested using 100 ng of target DNA and 10 pg of each primer. Species discrimination was assessed using *P. infestans* specific primers with *P. sojae* DNA and *P. sojae* primers with *P. infestans* DNA. The PCR amplification consisted of one cycle of 96 C for 2 min, followed by 30 cycles of 54 C (PiF plus PiR or PiF plus CBD-Rc), or 60 C (PsF plus PsR or PsF plus CBD-Rc) for 15 s and 72 C for 45 s, then a final cycle of 72 C for 5 min, using a Techne T-312 heated lid thermal cycler. Levels of detectable DNA were assessed by diluting *P. infestans* DNA (300 ng per microliter) to concentrations as low as 1 pg. An initial amplification used the primer pair PiF and PiR in 40 Âµl reactions. One microliter of the first amplification was used in a 40 Âµl second round amplification using the primer pair PiF and CBD-Rc. Samples were separated in 1% agarose gels. A comparison between CBD1 primers and Ypt1 primers was made using 10 pg of DNA from *P. sojae* and *P. infestans*.

## Results

### Isolation and Identification of Sequences Encoding CBD 1

**Fig. 1:**
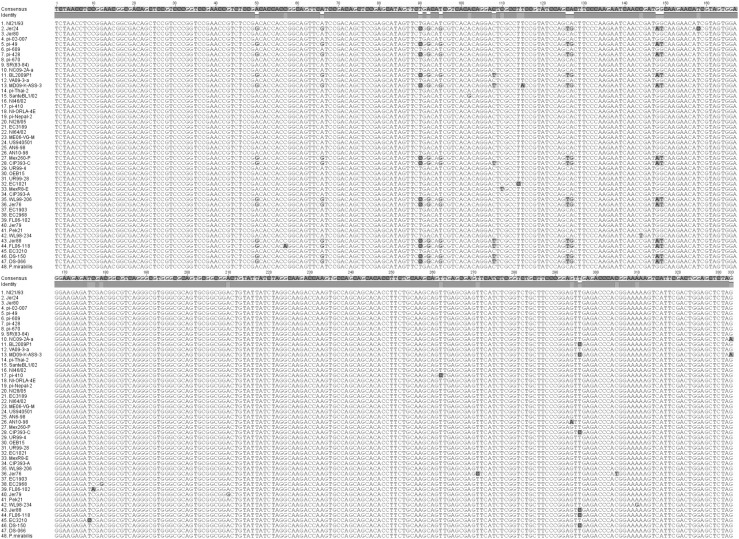
Nucleotide sequence alignment comparison of *Phytophthora infestans* isolates and *Phytophthora mirabilis* implemented in ClustalO 1.2.2 using default parameters. Output image generated using Geneious 8.1.7 (Color figure online)

Nucleotide alignments reveal the mature protein-coding regions that can be used in designing primers for detection of *P. infestans* using CBD1 (Fig. 1).

### Alignments and Phylogeny

**Fig. 2:**
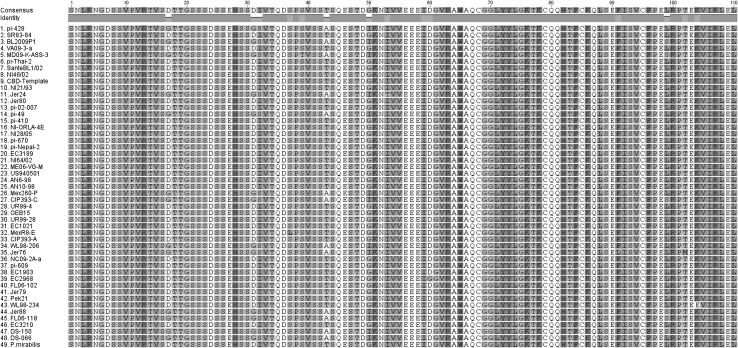
Amino acid sequence alignment comparison of *Phytophthora infestans* isolates and *Phytophthora mirabilis* implemented in ClustalO 1.2.2 using default parameters. Output image generated using Geneious 8.1.7. The mature protein is represented, with the signal peptide removed (Color figure online)

**Fig. 3:**
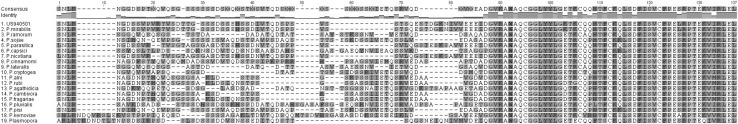
Amino acid sequence alignment comparison of *Phytophthora infestans* (isolate US940501) with 18 other species of *Phytophthora*, and *Plasmopara halstedii* implemented in Geneious 8.1.7/ClustalW (gap open cost:15; gap extended cost:6.66) (Color figure online)

**Fig. 4:**
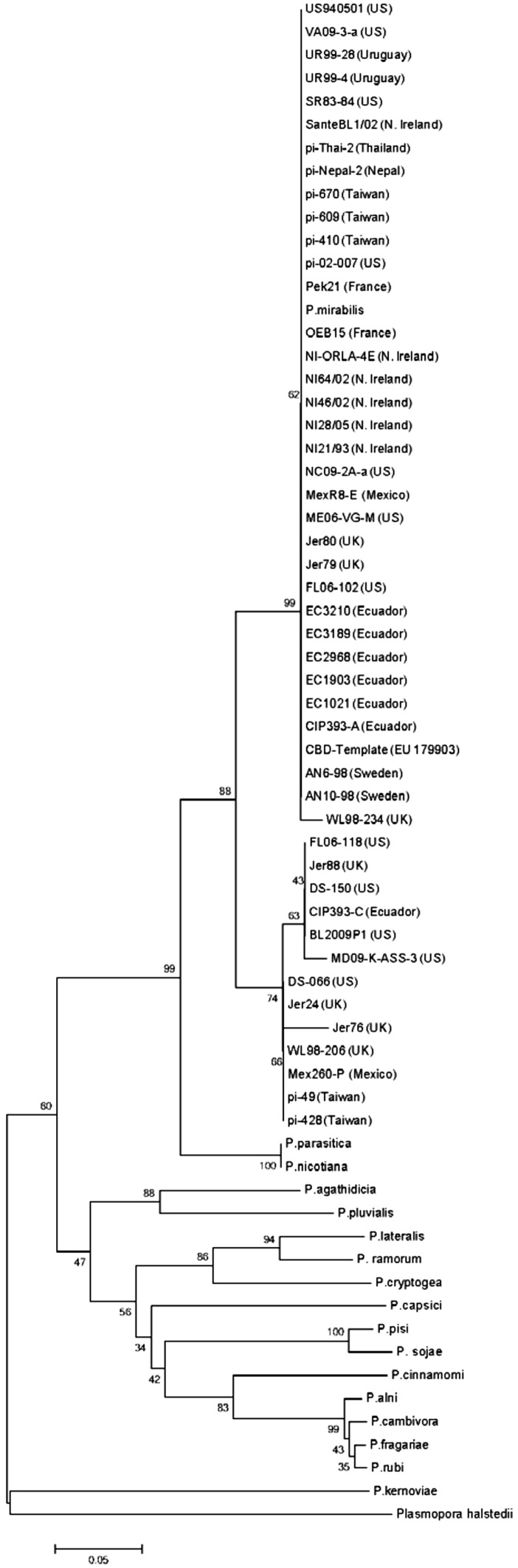
Phylogenetic analysis generated using the MEGA6 neighbor-joining method. Bootstrap values (500 replicates) are shown at the *nodes*. *Scale bar* represents evolutionary distances computed using the p-distance method (units being the number of amino acid substitutions per site)

While the *P. infestans* CBD1 amino acid sequence was highly conserved at the cellulose-binding-domain-containing carboxy half of the protein, there were a limited number of substitutions in the amino half of the secreted protein (Fig. 2), but these were mostly synonymous. Clear differences were seen in the amino terminus of the different *Phytophthora* species as well as *Plasmopara* (Fig. 3). Interestingly the carboxy terminal half was conserved up to the last amino acid in the protein. Distinct groups of isolates within *P. infestans*, and between *Phytophthora* species could be seen when assessing phylogeny based on amino acid sequences (Fig. 4).

### Application of CBD 1 Primers for Species Detection and Identification

Primer sets based on CBD1 sequence were able to discriminate between *P. infestans* and *P*. *sojae*. Primers targeting the full length sequence of the CBD-encoding gene, as well as a reverse primer within the conserved cellulose-binding domain allowed for species identification. Application of the terminal sequence primers followed by a nested reaction with the same forward primer and the cellulose-binding domain reverse primer allowed for the detection of *P*. *infestans* DNA concentrations of one picogram (Online Resource 2). When comparing CBD1 primers with Ypt1, the Ypt1 primers and the PiF CBD-Rc combination were able to detect 10 pg of DNA in a single reaction (Online Resource 3). The Ypt1 primers produced a stronger signal, but did not discriminate between *P. infestans* and *P. sojae*. Nested primers would be required to detect CBD1 as efficiently as Ypt1 using the primers from this study.

## Discussion

The highest degree of similarity within the *Phytophthora infestans* CBD 1 amino acid sequence was centered on the carboxyl half of the protein where the specific amino acids involved in cellulose binding are located. There were also a very limited number of differences on the amino terminal half of the protein, suggesting that region of the protein plays an important, but as yet undefined role. Variations in the amino terminal regions were random, except for a small group of *P. infestans* isolates that had an elevated level of amino acid substitution (5 aa changes). This group of isolates (FL06-118-US, Jer88-UK, DS150-US, CIP393C-Ecuador, BL2009P1-US, MD09KASS3-US, DS066-US, Jer24-UK, WL98206-UK, Mex260P-Mexico, PI49-Taiwan, and PI428-Taiwan) had an identical set of substitutions (D17/G, D31/G, I32/S, T43/A, and G50/A), suggesting that an original isolate was distributed to other countries, possibly through infected plant material. Sequence variations in CBD1 may prove a simple, useful tool to track the origins of other *Phytophthora* isolates.

At the species level the cellulose-binding domain continued to be conserved at the carboxy half of the protein, while the amino terminus was subject to larger variations occurring through insertions, deletions, and substitutions. If the amino terminal region interacts with other protein or carbohydrate components of the cell wall, then it is possible that CBD1 variations reflect the need to interact with the differing wall compositions found in different species [39, 43]. A large deletion (22 aa) is seen in *Plasmopara*, which differs from *Phytophthora* in being a strict biotroph. The presence of CBD1 in the downy mildew *Plasmopara* is the first evidence for this protein in downy mildews. The amino terminal region differs between *Phytophthora* and *Plasmopara* in potential for glycosylation. There are two predicted N-glycosylation sites (N8 and N13) in the *Plasmopara* CBD1-secreted protein and none in the *Phytophthora*-secreted CBD1, while there is one predicted O-glycosylation site (S8) in *Phytophthora* and none in *Plasmopara*. Glycosylation can allow for additional sites of carbohydrate interactions [14].

There are a wide range of gene targets for detecting and identification of *Phytophthora* species, including ITS regions, SSRs, and gene-specific primers [17, 21]. Primers spanning the introns of the vesicle transport-encoding Ypt1 gene were shown to be a reliable and efficient way to detect *P. nicotianae*, and these primers could also be used to distinguish some species of *Phytophthora* [5, 8, 30]. We were able to apply CBD1 nucleotide sequence information to design primers for the detection of *P. infestans* and *P. sojae*. One primer set was used to amplify the region of the CBD1 gene encoding the full secreted protein product. The second set that was tested amplified the genomic region from the beginning of the secreted protein to the highly conserved cellulose-binding domain region. Since this region is highly conserved, we used a common reverse primer that could be used for many different species of *Phytophthora*. The fact that *Phytophthora* contains at least three CBD proteins with a total of five cellulose-binding domains did not interfere with the use of the common domain primer sequence used from CBD1. While *Phytophthora* CBD-encoding genes are single copy, they are highly expressed during infection [1, 2]. Detection protocols based on RT-PCR could utilize CBD1-targeted primers and take advantage of the high transcript levels present in infected tissues.

In addition to their widespread occurrence in stramenopiles, cellulose-binding domains are found in non-enzymatic proteins of other organisms including yeast, slime molds, diatoms, and algae [18, 27]. In the case of *Phytophthora*, there is no evidence that the CBD-containing proteins move beyond the cell wall. They are not an abundant protein in cell wall proteomic analyses [29] yet they are readily detected by immunolocalization [18], suggesting that proteomic analysis may be missing tightly bound wall proteins. Cellulose-binding domains are of interest in biotechnological applications that include cellulose bioprocessing [4, 36, 44], protein purification tags [25, 31], and as modulators of plant growth [19, 23]. Information on the native variations within the cellulose-binding domain can provide insight into the degree of substitutions that can be generated without compromising function [16]. In a random selection of clones from five isolates we did not find allelic variation in CBD1. It is possible that allelic variants occur in CBD1, and this information may also add to our knowledge on the range of variation allowed without compromising function [11].

## Electronic supplementary material

Below is the link to the electronic supplementary material.

Online Resource 1. Identification of *Phytophthora infestans* isolate origins, and GenBank accession numbers for *Phytophthora* species and *Plasmopara* gene identification. Supplementary material 1 (PDF 37 kb)

Online Resource 2. PCR amplification of *P. infestans* and *P. sojae* DNA using CBD1 gene primers. Lane 1. Primers PiF and PiR plus 100 pg *P. infestans* DNA. Lane2. Primers PiF and CBD-Rc plus 100 pg *P*. *infestans* DNA. Lane 3. Primers PiF and PiR plus 100 pg *P. sojae* DNA. Lane 4. Primers PiF and CBD-Rc plus 100 pg *P. sojae* DNA. Lane 5. Primers PsF and PsR plus 100 pg *P. sojae* DNA. Lane 6. Primers PsF and CBD-Rc plus 100 pg *P. sojae* DNA Lane 7. Primers PsF and PsR plus 100 pg *P. infestans* DNA. Lane 8. Primers PsF and CBD-Rc plus 100 pg *P. infestans* DNA. Supplementary material 2 (PDF 56 kb)

Online Resource 3. Comparison of CBD1 and Ypt1 primers for detection of 10 pg of *P. infestans* (Pi) or *P. sojae* (Ps) DNA using PCR amplification. Primers used for CBD1 were PiF (TCTAACCTCCGGCGAC), PiR (TAGAGCTCCAGTCGAATGACT) PsF (TCCAACCTCCGAAACTCGATCCATC), PsR (TACAGCTCGAGCCGGATGACC) and CBD-Rc (GTGTGCTGCTGGCACTTGGTCT), which is a conserved region common across species of *Phytophthora*. The ras-related protein gene was targeted using Ypt1F (GACACGTACACGGAGAGCTACATCTCGACCAT) and Ypt1R (GTGCGGAAACGCTCCTGGCCGGC), based on common sequence from *P. infestans* (GenBank U30474) and *P. sojae* (GenBank XM_009517214). Lane 1. PiF and CBD-Rc. Lane 2. YptF and YptR. Lane 3. PsF and CBD-Rc. Lane 4. YptF and YptR. Lane 5. PiF and PiR. Lane 6. PsF and PsR. Supplementary material 3 (PDF 94 kb)
