# Supplementary material for: A Small Cellulose-Binding-Domain Protein (CBD1) in Phytophthora is Highly Variable in the Non-binding Amino Terminus
Source: Curr Microbiol. 2017 Jul 26;74(11):1287–93. doi: 10.1007/s00284-017-1315-x (PMC5640731; doi:10.1007/s00284-017-1315-x)
Supplement: Supplementary file 1 — Online Resource 1. Identification of Phytophthora infestans isolate origins, and GenBank accession numbers for Phytophthora species and Plasmopara gene identification. Supplementary material 1 (PDF 37 kb) [file 284_2017_1315_MOESM1_ESM.pdf]

A small cellulose binding domain protein (CBD 1) in Phytophthora is highly variable in the nonbinding amino terminus. Current Microbiology. R. Jones and F. Perez, USDA-ARS Beltsville, MD 20705. Richard.jones@ars.usda.gov

Table 1. Identification of isolates used to obtain CBD gene sequences. Isolates are maintained at the Beltsville International Phytophthora Collection.

Isolate	Country	Location
AN10-98	Sweden	Uppsala
AN6-98	Sweden	Uppsala
BL2009P1	USA	Pennsylvania
CIP393-A	Ecuador	
CIP393-C	Ecuador	
DS-066	USA	Florida
DS-150	USA	Florida
EC1021	Ecuador	
EC1903	Ecuador	
EC2968	Ecuador	
EC3189	Ecuador	
EC3210	Ecuador	
FL06-102	USA	
FL06-118	USA	
Jer 88	United Kingdom	Jersey Island
Jer24	United Kingdom	Jersey Island
Jer76	United Kingdom	Jersey Island
Jer79	United Kingdom	Jersey Island
Jer80	United Kingdom	Jersey Island
MD09-K-ASS-3	USA	Maryland
ME06-VG-M	USA	Maine
Mex260-P	Mexico	
MexR8-E	Mexico	
NC09-2A-a	USA	North Carolina
NI21/93	Northern Ireland	
NI28/05	Northern Ireland	
NI46/02	Northern Ireland	
NI64/02	Northern Ireland	
NI-ORLA-4E	Northern Ireland	
OEB15	France	
Pek21	France	
pi-02-007	USA	Michigan

pi-410	Taiwan	
pi-428	Taiwan	
pi-49	Taiwan	
pi-609	Taiwan	
pi-670	Taiwan	
pi-Nepal-2	Nepal	
pi-Thai-2	Thailand	
Sante BL1/02	Northern Ireland	
SR83-84	USA	Michigan
UR99-28	Uruguay	
UR99-4	Uruguay	
US940501	USA	New York
VA09-3-a	USA	Virginia
WL98-206	United Kingdom	Wales
WL98-234	United Kingdom	Wales

Table 2. Species identification and gene or genomic region harboring the CBD1 encoding gene.

Isolate	GenBank identifier
Phytophthora agathidicida	LGTR01000106
Phytophthora alni susp. alni	AUPN01239998
Phytophthora cambivora	AUVH01059526
Phytophthora capsici	LGTR01000106
Phytophthora cinnamomi	LGSK01000118
Phytophthora cryptogea	AUWJ02028117
Phytophthora fragariae	JHVZ03000053
Phytophthora kernoviae	AOFJ02000752
Phytophthora lateralis	AMZP02007804
Phytophthora nicotianae	LNFP010000335
Phytophthora parasitica	XM_008915048
Phytophthora pisi	CCEW01002060
Phytophthora pluvialis	LGTT01001527

Phytophthora ramorum	AAQX01000195
Phytophthora rubi	JMRJ01022618
Phytophthora sojae	XM_009538327
Plasmopara halstedii	CCYD01000645
