# Supplementary material for: A Small Cellulose-Binding-Domain Protein (CBD1) in Phytophthora is Highly Variable in the Non-binding Amino Terminus
Source: Curr Microbiol. 2017 Jul 26;74(11):1287–93. doi: 10.1007/s00284-017-1315-x (PMC5640731; doi:10.1007/s00284-017-1315-x)
Supplement: Supplementary file 2 — Online Resource 2. PCR amplification of P. infestans and P. sojae DNA using CBD1 gene primers. Lane 1. Primers PiF and PiR plus 100 pg P. infestans DNA. Lane2. Primers PiF and CBD-Rc plus 100 pg P. infestans DNA. Lane 3. Primers PiF and PiR plus 100 pg P. sojae DNA. Lane 4. Primers PiF and CBD-Rc plus 100 pg P. sojae DNA. Lane 5. Primers PsF and PsR plus 100 pg P. sojae DNA. Lane 6. Primers PsF and CBD-Rc plus 100 pg P. sojae DNA Lane 7. Primers PsF and PsR plus 100 pg P. infestans DNA. Lane 8. Primers PsF and CBD-Rc plus 100 pg P. infestans DNA. Supplementary material 2 (PDF 56 kb) [file 284_2017_1315_MOESM2_ESM.pdf]

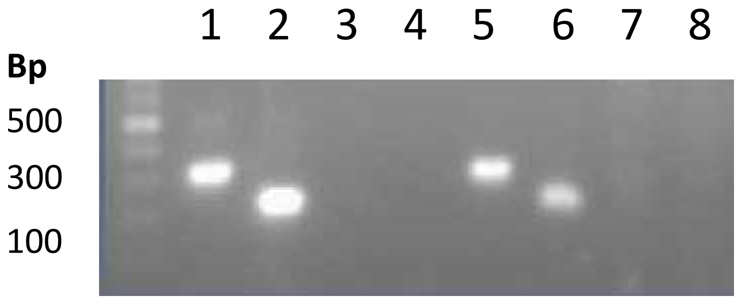

A small cellulose binding domain protein (CBD 1) in Phytophthora is highly variable in the nonbinding amino terminus. Current Microbiology. R. Jones and F. Perez, USDA-ARS Beltsville, MD 20705. Richard.jones@ars.usda.gov
