# Supplementary material for: A Small Cellulose-Binding-Domain Protein (CBD1) in Phytophthora is Highly Variable in the Non-binding Amino Terminus
Source: Curr Microbiol. 2017 Jul 26;74(11):1287–93. doi: 10.1007/s00284-017-1315-x (PMC5640731; doi:10.1007/s00284-017-1315-x)
Supplement: Supplementary file 3 — Online Resource 3. Comparison of CBD1 and Ypt1 primers for detection of 10 pg of P. infestans (Pi) or P. sojae (Ps) DNA using PCR amplification. Primers used for CBD1 were PiF (TCTAACCTCCGGCGAC), PiR (TAGAGCTCCAGTCGAATGACT) PsF (TCCAACCTCCGAAACTCGATCCATC), PsR (TACAGCTCGAGCCGGATGACC) and CBD-Rc (GTGTGCTGCTGGCACTTGGTCT), which is a conserved region common across species of Phytophthora. The ras-related protein gene was targeted using Ypt1F (GACACGTACACGGAGAGCTACATCTCGACCAT) and Ypt1R (GTGCGGAAACGCTCCTGGCCGGC), based on common sequence from P. infestans (GenBank U30474) and P. sojae (GenBank XM_009517214). Lane 1. PiF and CBD-Rc. Lane 2. YptF and YptR. Lane 3. PsF and CBD-Rc. Lane 4. YptF and YptR. Lane 5. PiF and PiR. Lane 6. PsF and PsR. Supplementary material 3 (PDF 94 kb) [file 284_2017_1315_MOESM3_ESM.pdf]

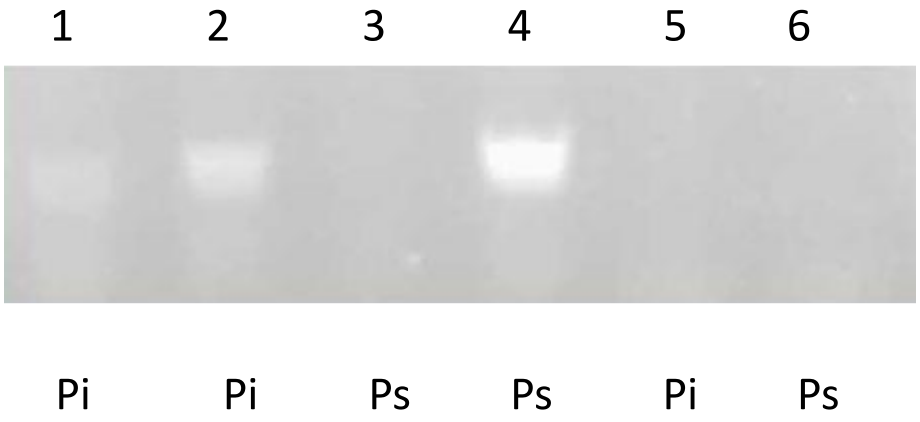

A small cellulose binding domain protein (CBD 1) in Phytophthora is highly variable in the nonbinding amino terminus. Current Microbiology.

R. Jones and F. Perez, USDA-ARS Beltsville, MD 20705.
Richard.jones@ars.usda.gov
